# Epigenomic signature of adrenoleukodystrophy predicts compromised oligodendrocyte differentiation

**DOI:** 10.1111/bpa.12595

**Published:** 2018-04-10

**Authors:** Agatha Schlüter, Juan Sandoval, Stéphane Fourcade, Angel Díaz‐Lagares, Montserrat Ruiz, Patrizia Casaccia, Manel Esteller, Aurora Pujol

**Affiliations:** ^1^ Neurometabolic Diseases Laboratory Bellvitge Biomedical Research Institute (IDIBELL), 08908 L'Hospitalet de Llobregat Barcelona Spain; ^2^ Center for Biomedical Research on Rare Diseases (CIBERER), ISCIII Spain; ^3^ Cancer Epigenetics and Biology Program (PEBC) Bellvitge Biomedical Research Institute (IDIBELL) Barcelona Catalonia Spain; ^4^ Department of Neuroscience and Neurology Icahn School of Medicine at Mount Sinai New York NY 10029; ^5^ Neuroscience Initiative ASRC CUNY, 85 St Nicholas Terrace New York NY 10031; ^6^ Physiological Sciences Department, School of Medicine and Health Sciences University of Barcelona (UB) Catalonia Spain; ^7^ Catalan Institution of Research and Advanced Studies (ICREA) Barcelona Catalonia Spain; ^8^ Present address: Biomarkers and Precision Medicine Unit (UByMP) Medical Research Institute La Fe (IISLaFE) Valencia Spain; ^9^ Present address: Translational Medical Oncology (Oncomet), Health Research Institute of Santiago (IDIS), University Clinical Hospital of Santiago (CHUS), CIBERONC Santiago de Compostela Spain

**Keywords:** adrenoleukodystrophy, epigenetics, myelin, neurodegeneration, oligodendrocytes, peroxisome

## Abstract

Epigenomic changes may either cause disease or modulate its expressivity, adding a layer of complexity to mendelian diseases. X‐linked adrenoleukodystrophy (X‐ALD) is a rare neurometabolic condition exhibiting discordant phenotypes, ranging from a childhood cerebral inflammatory demyelination (cALD) to an adult‐onset mild axonopathy in spinal cords (AMN). The AMN form may occur with superimposed inflammatory brain demyelination (cAMN). All patients harbor loss of function mutations in the ABCD1 peroxisomal transporter of very‐long chain fatty acids. The factors that account for the lack of genotype‐phenotype correlation, even within the same family, remain largely unknown. To gain insight into this matter, here we compared the genome‐wide DNA methylation profiles of morphologically intact frontal white matter areas of children affected by cALD with adult cAMN patients, including male controls in the same age group. We identified a common methylomic signature between the two phenotypes, comprising (i) hypermethylation of genes harboring the H3K27me3 mark at promoter regions, (ii) hypermethylation of genes with major roles in oligodendrocyte differentiation such as *MBP*, *CNP*, *MOG* and *PLP1* and (iii) hypomethylation of immune‐associated genes such as *IFITM1* and *CD59*. Moreover, we found increased hypermethylation in CpGs of genes involved in oligodendrocyte differentiation, and also in genes with H3K27me3 marks in their promoter regions in cALD compared with cAMN, correlating with transcriptional and translational changes. Further, using a penalized logistic regression model, we identified the combined methylation levels of *SPG20*, *UNC45A* and *COL9A3* and also, the combined expression levels of *ID4* and *MYRF* to be good markers capable of discriminating childhood from adult inflammatory phenotypes. We thus propose the hypothesis that an epigenetically controlled, altered transcriptional program may drive an impaired oligodendrocyte differentiation and aberrant immune activation in X‐ALD patients. These results shed light into disease pathomechanisms and uncover putative biomarkers of interest for prognosis and phenotypic stratification.

## Introduction

X‐linked adrenoleukodystrophy (X‐ALD: McKusick no. 300100) is a neurometabolic genetic disorder characterized by progressive demyelination within the central nervous system (CNS), axonopathy in spinal cords and adrenal insufficiency. It is the most common monogenic leukodystrophy and peroxisomal disorder and has a minimum incidence of 1 in 17 000 males. This disease is caused by mutations in the ABCD1 (ALD) gene (Xq28) encoding the peroxisomal ABC transporter 17, 53, which transports very‐long‐chain fatty acids (VLCFAs) or VLCFA–CoA esters into the peroxisome for degradation by β‐oxidation 75. Three major disease variants have been described: a late‐onset form affecting adults, called adrenomyeloneuropathy (AMN) because it exhibits peripheral neuropathy and distal axonopathy in the spinal cord with secondary demyelination—but no brain demyelination—with spastic paraparesis as the major symptom, and two ultimately lethal forms with cerebral demyelination and neuroinflammation: an adult form called cAMN and an acute, childhood cerebral form called cALD. Evolution of disease in the cerebral forms is very fast and inexorable in most cases. In contrast, AMN is a chronic, slowly progressive form and relapsing‐remitting presentations are never seen, unlike in multiple sclerosis subtypes 35, or rarely, in other leukodystrophies such as metachromatic leukodystrophy 2. The clinical course combined with the lack of descriptions of remyelination in neuropathological or MRI studies, may indicate hampered regeneration capacities of the brain myelin, owing to reasons not currently understood 21. Interestingly, all clinical phenotypes can occur within the same family and be even discordant in monozygotic twins 37, 70, illustrating a lack of phenotype–genotype correlation adding a layer of complexity to an in principle, monogenic disorder, and thus suggests the participation of modifying genetic factors, stochastic, environmental, or epigenetic events in the variable disease expressivity in X‐ALD 17.

All X‐ALD patients accumulate saturated VLCFAs and to a lesser extent, monounsaturated VCLFAs in plasma, peripheral blood and tissues, most notably in the brain and adrenal cortex 17, 52. Excess VLCFA results in increased free radical production from mitochondria and altered cellular proteostasis 22, 38, 39, 45. Additionally, VLCFAs are incorporated into complex lipids within cell membranes and are thought to destabilize myelin sheaths by occupying the lateral chains of proteolipid proteins, gangliosides and phospholipids 8, 68. Both the structural disturbances caused by excess of VLCFA and the impact of this excess in essential functions such as redox and energetic dyshomeostasis, or protein turnover may account for the axonal degeneration and myelin demise in this disease 18. Although disease severity may correlate with increased VLCFA content in the white matter 4, it remains to be determined whether excess VLCFAs may trigger cerebral inflammation and demyelination. Indeed, a robust inflammatory response occurs in the brain white matter in cALD patients, whereas minimal or no inflammatory lesions have been reported in tissues from pure AMN patients 52. Thus, additional pathogenic factors may explain the panoply of clinical manifestations in X‐ALD. The identification of these factors is an outstanding question in the field that may guide informed prognosis and improved disease management, given that the available therapies for cALD, such as hematopoietic stem cell transplantation and hematopoietic stem cell gene therapy, exhibit a narrow therapeutic window. Remyelination does not occur in X‐ALD, in contrast to other demyelinating diseases such as the relapsing‐remitting forms of multiple sclerosis. This is the case even after successful hematopoietic stem cell transplantation, efficacious at halting disease progression only. The reasons for this lack of regeneration are not currently understood.

Epigenetics involves heritable changes in gene expression not derived from DNA sequence and includes DNA methylation, a variety of posttranslational modifications to histones and microRNA 5. Although the study of DNA methylation in cancer has been a paradigmatic model for its role in regulating expression of tumor suppressors and oncogenes, a significant role of DNA methylation in neurological disorders has also been reported. Notable examples include schizophrenia, multiple sclerosis and Alzheimer's disease, thus suggesting the association of subtle changes in DNA methylation with pathogenic processes 32, 74. Clinical and preclinical studies have further supported the concept that epigenetic dysregulation of DNA methylation and histone acetylation patterns are associated with inflammation and demyelination 25.

Within this conceptual framework, we set out to investigate whether differential DNA methylation changes in the brains of X‐ALD patients might contribute to explaining the lack of a genotype‐phenotype correlation. We thus compared the genome‐wide methylation profiles of intact brain white matter tissue from X‐ALD patients with distinct phenotypes with those from healthy age and sex‐matched controls and uncovered differential methylation in genes involved in oligodendrocyte differentiation and the immune response. Validation of these methylation changes by pyrosequencing, qRT‐PCR and western blotting revealed a novel X‐ALD disease signature.

## Material and Methods

### Human brain samples

White matter tissue samples from the prefrontal cortex area of cALD or cAMN patients and healthy age male control in the same age group, were obtained from the NICHD Brain and Tissue Bank for Developmental Disorders at the University of Maryland, Baltimore, MD, USA, as previously described. Briefly, frozen blocks of normal‐appearing white matter were dissected from frontal lobes from 8 child and 9 adult controls and 8 child cALD and 9 adult cAMN patients. All the children and adults with cerebral ALD had the conventional “parieto‐occipital” form of cerebral ALD. White matter sections of ALD patients and controls were stained with Luxol Fast Blue (LFB) to detect demyelination, identify the demyelination edge and the normal‐looking intact area. Brain tissue sections were processed when two to three adjacent sections showed no sign of demyelination with LFB staining and no perivascular cuffs of lymphocytes using hematoxylin and eosin staining 62. Informed written consent was obtained from all patients or their legal representatives, and the institutional ethics committee approved the studies (Supporting Information Table S1).

### Microarray‐based DNA methylation analysis

Genome‐wide DNA methylation analysis was performed using the Infinium Human Methylation 450 BeadChip from Illumina. The 450K DNA methylation array by Illumina is an established, highly reproducible method for DNA methylation detection and has been validated 60.

DNA was isolated and subjected to sodium bisulfite treatment to generate methylation‐specific base changes before hybridization. Batch effects were minimized by randomized placement of samples from X‐ALD patients and controls across the arrays. Potential false positives were controlled for by removing probes with low signal intensity (detection *P* value > 0.01) and those that overlapped with common SNPs. Methylation values for individual CpG sites in each sample were measured as β‐values, which represent the ratio of the methylated hybridization signal intensity to the sum of both methylated and unmethylated signals after background subtraction. To assess differences in methylation between groups, the original ∼485 000 β‐values were converted to *M*‐values via the logit transformation, as recommended by Du *et al* 13. The β‐value has a more intuitive biological interpretation, but the *M*‐value is more statistically valid for the differential analysis of methylation levels. Therefore, we used the *M*‐value method for conducting differential methylation analysis and included the β‐value when reporting the results in tables and figures.

DNA from patients was quantified with Quant‐iT™ Pico Green dsDNA Reagent (Invitrogen), and the integrity was analyzed in a 1.3% agarose gel. Bisulfite conversion of 600 ng of each DNA sample was performed according to the manufacturer's recommendations for the Illumina Infinium Assay. Effective bisulfite conversion was verified for three controls that were converted simultaneously with the samples; 4 µl of bisulfite‐converted DNA was used for hybridization on to an Infinium Human Methylation 450 BeadChip, according to the Illumina Infinium HD Methylation protocol. Chip analysis was performed using an Illumina HiScan SQ fluorescence scanner.

### Data normalization and methylation status analysis

Epigenetic (DNA methylation) status was defined using genome‐wide DNA methylation analysis with Infinium Human Methylation450 BeadChips from Ilumina. The 450K DNA methylation array includes 485 764 cytosine positions of the human genome. The intensities of the images were extracted and normalized using the Genome Studio (2011.1) Methylation module (1.9.0) software. The methylation score of each CpG is represented as the beta (β) value.

For determining differential methylation, β‐values were first converted to *M*‐values via the logit transformation, as recommended by Du *et al* 13. Then, differentially methylated probes were detected using the Limma package 69. The Limma procedure uses linear models to assess differential methylation whereby information is shared across probes. A major benefit of the Limma procedure is that it allows for factorial analysis in the specification of the linear model. As such, we were able to adjust for age in the detection of differentially methylated probes between phenotypes by including two‐levels: children‐adult in the specification of the Age factor in the design matrix. Samples were easily divided into two age groups: children, comprising patients from 4.5 to 13 years of age, and adults, comprising patients from 35 to 68 years of age. Therefore, for cALD and cAMN phenotype differences, the design matrix specified only one contrast [(ALD.child‐CTL.child) – (ALD.adult‐CTL.adult)], characterizing the genotype‐by‐age interaction between X‐ALD and controls in children and adults. For differences in X‐ALD compared with controls common to both phenotypes, we used a one‐factor genotype design. In both approaches described above, *P* values were adjusted across genes using Benjamini–Hochberg correction.

### Pyrosequencing‐based DNA methylation validation

Pyrosequencing analyses to determine CpG methylation status were developed as previously described 61. Briefly, a set of primers for PCR amplification and sequencing were designed using a specific software pack (PyroMark assay design version 2.0.01.15). Primer sequences were designed to hybridize with CpG‐free sites to ensure methylation‐independent amplification. PCR was performed with primers biotinylated to convert the PCR product to single‐stranded DNA templates. We used the Vacuum Prep Tool (Biotage, Sweden) to prepare single‐stranded PCR products according to the manufacturer's instructions. Pyrosequencing reactions and methylation quantification were performed with a PyroMark Q96 System version 2.0.6 (Qiagen). Graphic representation of methylation values for each region were obtained from the averages of the CpG dinucleotides included in the sequence analyzed, and a non‐parametric Mann–Whitney *U* test was used to test for significance.

### Quantitative RT‐PCR

To use an orthogonal approach to the integrated biology (‐omic) methods, total RNA from PBMCs was extracted using an RNeasy Kit (Qiagen) according to the manufacturer's instructions. One microgram of RNA was transcribed into cDNA using Superscript II reverse transcription reagents in a final volume of 25 μL (Invitrogen). Gene expression was measured using 0.1–0.2 μL of cDNA. TaqMan real‐time PCR was performed with an ABI PRISM 7300HT sequence detection system using TaqMan^®^ Universal PCR master mix and standardized primers for *ID4* (Hs02912975_g1), *NINJ2* (Hs00356576_m1), *OPALIN* (Hs01088845_m1), *MYRF* (Hs00973721_g1), *MBP* (Hs00921945_m1), *MOG* (Hs00159219_m1), *CNP* (Hs00263981_m1), *PLP1* (Hs00166914_m1), *SOX2* (Hs01053049_s1), *IFITM1* (Hs00705137_s1), *UNC45A* (Hs00218751_m1), *OLIG1* (Hs00744293_s1) and *LPIN1* (Hs01041902_m1). Expression of the genes of interest was normalized to that of the reference control, human *RPLP0* (Hs99999902_m1). Each sample was run in duplicate, and the mean value of the duplicates was used to calculate the mRNA expression using the comparative (2^−ΔCt^) method, according to the manufacturer's instructions 50.

### Antibodies and reagents

Antibodies used for western blots were purchased from the following commercial sources: anti‐H3K27me3 [Diagenode (C15320069); dilution: 1:1000], anti‐H3K9me3 [Millipore (07‐442); dilution: 1:1000], anti‐H3 [Cell Signalling (9715), dilution: 1:1000] and goat anti‐rabbit and goat anti‐mouse IgG conjugated to horseradish peroxidase (DakoCytomation, dilution: 1:15000).

### Immunoblot analysis

Tissue samples were lysed in ice‐cold RIPA buffer (50 mM Tris‐HCl, pH 8, 12 mM deoxycholic acid, 150 mM NaCl and 1% NP40, supplemented with Complete Protease Inhibitor Cocktail and anti‐phosphatases, Roche) using a Teflon‐on‐glass homogenizer and then centrifuged at 7000 g for 10 min at 4°C. The protein concentration was determined using a BCA protein assay kit (Thermo Fisher Scientific, Inc.). Samples (30 μg) were boiled for 5 min in Laemmli's buffer and run on Bis‐Tris gels with a Bolt^TM^ mini gel tank (Invitrogen) using MOPS SDS running buffer Bolt^TM^ (Invitrogen). After electrophoresis, proteins were transferred to nitrocellulose membranes using an iBlot^®^2 gel transfer device (Invitrogen) for blotting with appropriate antibodies. Proteins were visualized with an enhanced chemiluminescence western blot detection system (GE Healthcare Bio‐Sciences AB), after exposure to CL‐XPosure Film (Thermo Scientific).

### Statistical analysis

The positional distribution of probes within DMRs with respect to CpG feature and RefSeq genes was compared with the overall distribution of all filtered probes on the array, and enrichment *P* values were determined with Fisher's exact test. To evaluate which pathways or functional categories were enriched in differentially methylated genes, we computed a hypergeometric distribution with Benjamini–Hochberg Multiple Testing Correction in the Molecular Signatures Database (MSigDB) data set. To evaluate the difference in β‐value levels between cALD and cAMN, we performed a two‐tailed paired t‐test between the CpG mean β‐value over 0.1 or below −0.1 in cALD and cAMN for each CpG after age correction in all differential DMRs or in a given specific enriched functional set. To correct for the effect of age we did the following: from the CpG mean values of cALD and cAMN we subtracted the CpG mean intensities of control child and control adult, respectively. Pyrosequencing, Q‐PCR array expression and WB data were examined for normality with the Shapiro–Wilk test. Significant differences were determined using Anova (when a factor with more than 2 levels) or a two‐tailed Student's *t*‐test if the data were normally distributed or two‐tailed Kruskal‐Wallis rank sum test (when a factor with more than 2 levels) or the Wilcoxon rank sum test otherwise.

All penalty regression methods in this study were conducted using the glmnet package in the statistical software R. The “cv.glmnet” function incorporates a number of model‐fitting practices including n‐fold cross validation and Least Absolute Shrinkage and Selection Operator (LASSO) regularization to decrease model overfitting. The Lasso penalty parameter was selected using the automatic cross‐validation. By default, our method used a leave‐one‐study‐out crossing validation, where the n in n‐fold corresponds to the number of samples in our experiment.

The power to discriminate cALD and cAMN using methylation CpGs values and gene expression data was evaluated by calculating the area under the curve (AUC) of the receiver operating characteristic (ROC) curve. The pROC package in R was used to calculate AUCs along with their standard errors and 95% confidence intervals 58. The DeLong test was used to compare the areas under two different correlated ROC curves. To rule out the variable “age” as a confounding factor affecting the results, we applied the CpG and qPCR models independently in adult and child controls. In these control samples, the CpGs and expression models were not able to discriminate between adults and children, thus underscoring the robustness of the approach to discriminate between cALD and cAMN independently of the variable “age.” All statistical analyses were performed using Bioconductor packages in an R programming environment.

### Data availability

Data that support the findings of this study have been deposited in Gene Expression Omnibus with the accession code GSE78218. Expression microarray data referenced in this study are available in Array Express Database with the accession code E‐MEXP‐3288 (62). All other relevant data are available from the corresponding author.

## Results

### Global DNA methylation analysis of X‐ALD brains

**Figure 1:**
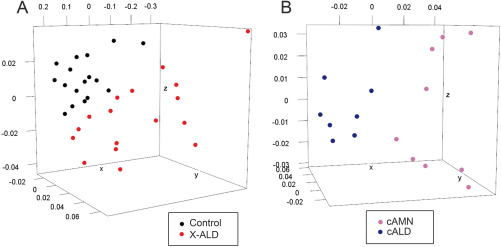
*Three‐dimensional multidimensional scaling (MDS) plot of methylation levels in β‐values, which is a method measuring the intensities of the methylation, ranging from 0 to 1, from differential CpGs*. In (**A**), common methylation differences in 17 child and adult X‐ALD samples with respect to those in 17 controls, and in (**B**), methylation differences between 8 cALD and 9 cAMN samples. We used the RGL package for 3D visualization.

We chose to analyze samples of intact brain white matter of prefrontal cortex, owing to the tissue specificity of epigenetic changes and selected areas lacking inflammatory infiltrates or other abnormalities after a thorough neuropathological examination as previously described, see Material and Methods 62. These white matter samples were obtained from the prefrontal cortex area of 8 child and 9 adult cerebral X‐ALD subjects and of 8 child and 9 adult controls (Supporting Information Table S1). We sought to determine the genome‐wide distribution of DNA methylation in normal children and adults, compare it with that in patients with a diagnosis of cerebral X‐ALD (cALD and cAMN) and define the existence of potential elements of distinction. We first assessed the occurrence of genome‐wide methylation changes occurring in all X‐ALD patients compared with controls (ie, grouping childhood and adult patients altogether). We then defined a two‐factorial experiment with genotype and age factors to adjust for the detection of differentially methylated probes between the cALD child and cAMN adult samples. To determine whether the DNA methylation values in the X‐ALD and control samples segregated independently, we performed multidimensional scaling (MDS) using β‐values, which is a method measuring the intensities of the methylation at the interrogated CpG sites, ranging from 0 to 1, of the statistically significant CpGs (*P* < 0.01). The MDS revealed a clear separation between X‐ALD samples and controls (Figure 1A) and between cALD and cAMN cases (Figure 1B).

The majority of both hypomethylated and hypermethylated CpGs revealed subtle changes within the range of modifications consistent with reported environment‐gene interactions 32, 71. Because regulatory methylation changes generally encompass multiple CpGs, we sought to identify regions of the genome with differential DNA methylation between X‐ALD disease and control samples, rather than focusing on isolated changes at individual CpGs. To that end, we used a 1‐kb sliding window analysis, a variation of the tiling regional analysis that has been optimized for DNA methylation analysis, as previously reported 32. Differentially methylated regions (DMRs) were defined as those windows showing statistical significance after false discovery rate (FDR) correction. To identify genes associated with each DMR when a given gene could not be assigned, we overlapped the DMR with DNase I‐hypersensitive sites (DHSs) with significant correlations with the expression levels of nearby genes in 112 ENCODE cell lines 64.

**Figure 2:**
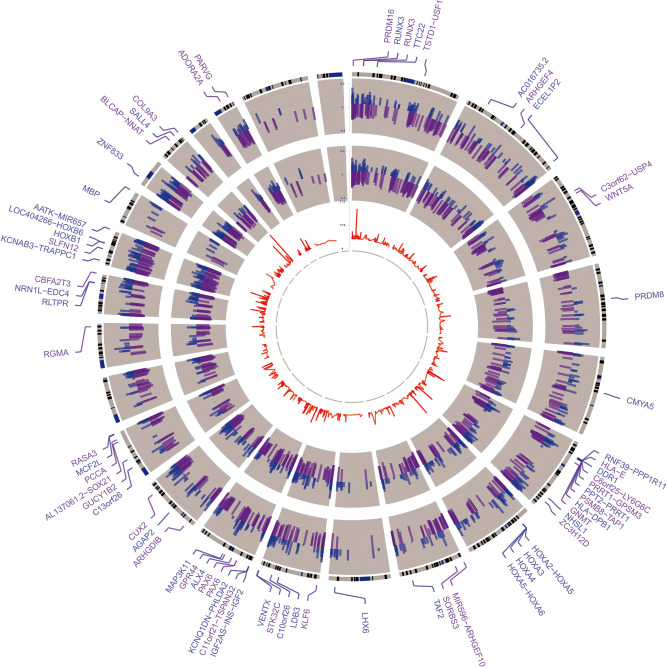
*Circos plot of common genome‐wide DNA methylation changes in cALD and cAMN brains of X‐ALD patients with respect to methylation in controls*. The outermost ring shows the RefSeq genes associated with differentially methylated DMRs: genes associated with hypomethylated and hypermethylated DMRs are shown in purple and blue, respectively. The second circle represents genome positions according to chromosome (black lines are cytobands). The third and fourth circles represent the β‐value difference between X‐ALD and controls in the same age group for significant DMRs in cALD and cAMN samples, respectively. Blue lines signify hypermethylated regions, and purple lines signify hypomethylated regions, with the length of each line representing the difference level. Each line also marks the location of the Illumina 450K probe distribution along the genome. The innermost red line represents the best Fisher's method −log10(*P* value) per 1‐kb window analyzed within the merged DMR represented. For a clear representation, only gene names with an FDR *P* value <1 E – 10 are shown. Circular plots were drawn with the software application OmicCircos 29.

**Figure 3:**
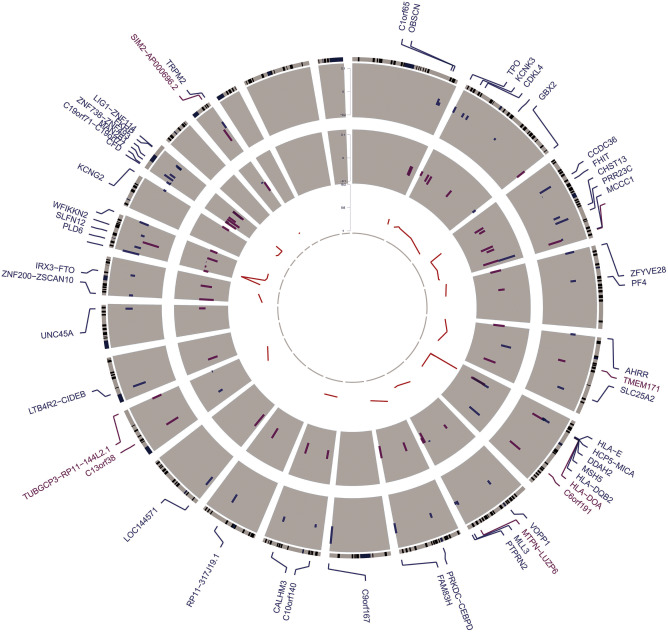
*Circos plot of genome‐wide DNA methylation differences between cALD and cAMN brains of X‐ALD patients*. The outermost ring shows the RefSeq genes associated with differentially methylated DMRs: genes associated with hypomethylated and hypermethylated DMRs are shown in purple and blue, respectively. The second circle represents genome positions by chromosomes (black lines are cytobands). The third and fourth circles represent the β‐value difference between cALD and cAMN after age correction for significant DMRs, respectively. Blue lines signify hypermethylated regions, and purple lines signify hypomethylated regions, with the length of each line representing the difference level. Each line also marks the location of the Illumina 450K probe distribution along the genome. The innermost red line represents the best Fisher's method −log10(*P* value) per 1‐kb window analyzed within the merged DMR represented. Circular plots were drawn with the software application OmicCircos 29.

In the first analysis, we identified 4632 DMRs (containing 10243 differentially methylated CpGs) that were statistically significant (at *P* < 0.0001 after FDR correction) in cerebral X‐ALD samples compared with control samples. Overlapping regions were merged to form a non‐redundant set of 1513 DMRs. In a second‐level analysis, we sought to pinpoint the methylomic differences between cALD and cAMN after correcting for the age factor. This comparison identified 172 DMRs (containing 433 CpGs) that were differentially methylated in cALD and cAMN brain samples (*P* value of *P* < 0.01 after FDR correction). Overlapping regions were merged to form a non‐redundant set of 56 DMRs. The genome‐wide distribution of all DMRs is represented as a circular ideogram composed of concentric circles depicting the methylation levels in cALD and cAMN compared with those in the same age group controls, with chromosomal locations annotated in a clockwise manner and statistical significance indicated by radial arrangements and color codes (Figure 2). Figure 2 depicts the differential methylomic profiles of both phenotypes of X‐ALD compared with those of controls, that is, the methylomic profile that was similar between cALD and cAMN samples but different from controls and therefore defined a “disease‐specific signature.” In Figure 3, we show the different age‐corrected methylomic profiles between cALD child and cAMN adult patients resulting from the 2‐factor analysis, thereby focusing on divergent methylation trends.

**Figure 4:**
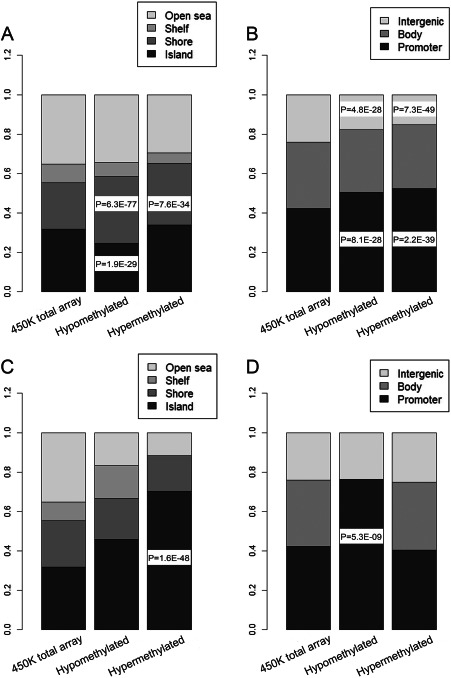
*CpG distribution in X‐ALD patients*. Distribution of CpG in islands, shores, shelves and open sea (**A**, **C**) relative to RefSeq gene promoters, gene bodies and intergenic regions (**B**, **D**) in X‐ALD samples with respect to that in controls (A, B) and in cALD samples with respect to that in cAMN (C, D). CpGs in hypomethylated and hypermethylated DMRs are compared with CpGs on the total Illumina array using Fisher's exact test.

We next compared the distribution of hypomethylated and hypermethylated DMRs with the CpG distribution in the Illumina 450K array (Figure 4). Hypomethylated DMRs in samples from X‐ALD patients were preferentially found in CpG shores rather than islands (*P* = 6.3 E – 77; Figure 4A). Hypermethylated DMRs were also preferentially distributed in shores rather than shelves or open sea regions (*P* = 7.6 E – 34; Figure 4A). Notably, the overall distribution of the DMRs relative to RefSeq genes revealed a significant enrichment in hypomethylated and hypermethylated CpGs in promoters, with *P* = 8.1 E – 28 and *P* = 2.2 E – 39, respectively (Figure 4B). We also analyzed the DMR methylation distribution in child cALD vs. adult cAMN patients. DMRs in samples from child cALD patients were preferentially located at CpG islands rather than shores, with *P* = 1.6 E – 48 in DMRs hypermethylated (Figure 4C). Hypomethylated CpGs in childhood cALD relative to adult cAMN were preferentially distributed within promoters, with *P* = 5.3 E – 09 (Figure 4D). We did not identify any hypomethylated DMRs in the gene bodies among the 433 differential DMRs between cALD and cAMN. Thus, differential methylation between all X‐ALD samples and controls showed an overall enriched distribution at CpG shores and islands within gene regulatory regions, thereby suggesting that these methylations may have functional consequences in gene expression 12. It is worth mentioning that the values obtained from the 450K analysis account for levels of 5‐methylcytosine and 5‐hydroxymethylcytosine (5 hmc). The latter may also constitute a stable epigenetic modification shown to contribute regulatory functions to the epigenome 9. This, however, has been related to a relative enrichment of 5 hmc levels in the gene body when compared to promoter regions (defined as within 1500 bp upstream of transcriptional start site) 72. This scenario does not seem to apply to X‐ALD as we do not find an enrichment of total methylation levels in gene bodies.

**Figure 5:**
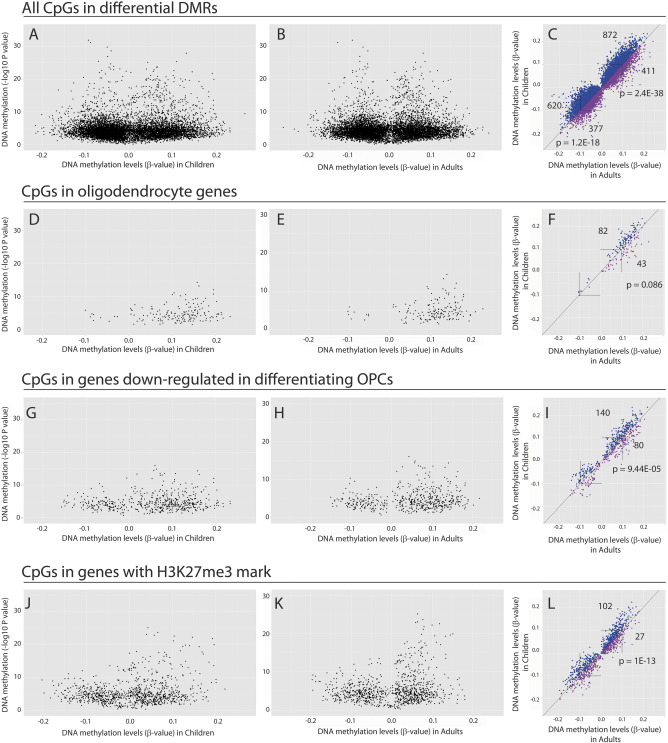
*Volcano plot of DNA methylation levels for all CpGs and X‐ALD signature CpGs‐associated genes within DMRs identified by sliding window analysis in brains of X‐ALD and controls*. The dot marks the CpG mean β‐value difference between childhood (**A**, **D**, **G** and **J**) and adult (**B**, **E**, **H** and **K**) X‐ALD with controls for significant windows. Each dot also represents the Fisher's method −log10(*P* value) for each 1‐kb window analyzed. A, B and **C**, plots display all CpGs within differential DMRs. D, E and **F**, plots display CpG‐associated genes enriched in oligodendrocytes in the adult mouse. G, H and **I**, plots display CpG‐associated genes downregulated during differentiation of Oli‐Neu cells (oligodendroglial precursor, OPCs) in response to PD174265. J, K and **L** plots display CpG‐associated genes with the trimethyl H3K27 (H3K27me3) mark. In C, F, I and L, plots represent the CpG mean β‐value in cALD with respect to that in cAMN. A blue dot indicates a CpG more methylated in cALD, and a red dot indicates a CpG more methylated in cAMN. The number of CpGs more methylated with a β‐value above 0.1 or below −0.1 with respect to that in the other phenotype is also indicated. The *P* value was computed using a paired *t*‐test between the CpG mean β‐value over 0.1 or below −0.1 in cALD and cAMN for each CpG within a differential DMR after age correction.

To begin addressing the functional signature of the widespread changes in DNA methylation, we performed an enrichment analysis using the Molecular Signatures Database (MSigDB) data set collected from various sources such as online pathway databases including KEGG, BioCarta, Reactome and publications in PubMed, which revealed distinct functional categories for common and differential methylation in child and adult X‐ALD DMR‐associated gene lists (Supporting Information Table S2‐S4). Figure 5 defines the CpGs within differential DMRs between X‐ALD and controls (Figure 5A–C) or in DMR‐associated genes for those enriched sets identified in the MSigDB analysis. We observed a clear hypermethylation of genes associated with oligodendrocyte lineage differentiation in both cALD and cAMN samples (Supporting Information Table S2). In particular, we identified hypermethylated DMRs in oligodendrocyte‐specific genes according to the gene set characterized in adult mouse brain oligodendrocytes 40 (Figure 5D–F). We also identified an enrichment in genes downregulated during differentiation of oligodendroglial precursor cells (Oli‐Neu cells) in response to the OPC differentiating agent PD174265 24 (Figure 5G–I). In addition, we identified hypermethylation of genes with the trimethylated H3K27 mark (H3K27me3) in their promoters in human embryonic stem cells. This process has been implicated in the restriction of multipotentiality and gene inactivation in stem cells 7 (Figure 5J–L). However, the hypomethylated DMRs were detected in genes with promoters occupied by the PML‐RARA fusion protein in acute promyelocytic leukemia (APL) cell lines, on the basis of Chip‐seq data 48, and in genes that form the macrophage‐enriched metabolic network (MEMN) and have a causal relationship with metabolic syndrome traits 11 (Supporting Information Table S3). Together, these data revealed hypermethylation of genes implicated in oligodendrocyte function, and hypomethylation of immune‐related genes in both childhood and adult X‐ALD phenotypes compared with their respective controls.

We next compared the methylation patterns of X‐ALD brains against a specific genome‐wide methylation map of the transition from oligodendrocyte precursor cells to myelinating oligodendrocytes (OPC to OL). The comparison indicated that the X‐ALD methylome was enriched in genes identified as hypermethylated (*P* = 4.54 E – 37) and hypomethylated (*P* = 5.33 E – 27) during the OPC to OL transition as follows: (i) 68% (143 out of 209) of the hypermethylated genes in X‐ALD human brains represented those that are hypomethylated during differentiation of murine OPCs into OLs; (ii) 51% (108 out of 221) of the hypomethylated genes in X‐ALD brain samples represented those that are hypermethylated during the transition of murine OPCs into OLs. This reciprocal pattern of methylation suggested a profound alteration in the physiological mechanism of methylation‐driven repression necessary for OL differentiation (Supporting Information Table S5). An illustrative example is that of myelin genes, such as myelin oligodendrocyte glycoprotein (*MOG*), 2′,3′‐cyclic nucleotide 3′ phosphodiesterase (*CNP*), proteolipid protein 1 (*PLP1*), myelin basic protein (*MBP*), myelin‐associated oligodendrocyte basic protein (*MOBP*) or myelin associated glycoprotein (*MAG*), which were hypomethylated in OLs and were found to be hypermethylated in X‐ALD, both in the childhood and adult phenotypes (Supporting Information Table S5)

Because methylation of nucleosomal lysine residues at position K27 and K9 in Histone H3 has previously been shown to be involved in oligodendrocyte differentiation 42, 43, we overlapped our results with those for genes with H3K27me3 and H3K9me3 marks identified during OL differentiation in the rat brain 42. Thus, in this later work, the gene repression program of immature iOLs is mostly orchestrated by the deposition of the repressive H3K9me3 mark, whereas the H3K27me3 mark plays a role in repression at the progenitor state 42. In line with these findings, we found in X‐ALD brains a significant enrichment (*P* = 7.14 E – 06) in genes with the deposition of the repressive H3K27me3 mark in OPCs and an enrichment in genes with the H3K9me3 mark in immature oligodendrocytes (*P* = 2.41 E – 05). Again, these data support the enrichment in coexisting populations of OPCs and iOLs in X‐ALD brains of both childhood and adult phenotypes to the detriment of myelinating OLs.

### Methylomic differences between cALD and cAMN phenotypes

Seeking differential elements between children and adults with X‐ALD, we also examined the enrichment on the basis of the Molecular Signatures Database (MSigDB) data set in differentially methylated DMR‐associated gene lists between cALD samples and cAMN samples (Supporting Information Table S4). The results indicated an overrepresentation of several immune response pathways such as “diabetes,” “autoimmune thyroid disease,” “IL22 signaling,” and “eicosanoid receptors,” particularly with the involvement of the *HLA‐DOA, PTPRN2, HLA‐E, TPO, CEBPD, CFD, LTB4R* and *LTB4R2* genes in the childhood cALD samples. Thus, these data showed a differential methylation of immune‐related genes between cALD and cAMN that may be useful in discriminating one phenotype from another.

We next investigated whether the methylation grade was associated with disease severity or onset by comparing DNA methylation levels within differential DMRs between cALD and cAMN, using a paired t‐test. We found a significant hypermethylation on the basis of the mean β‐value for each CpG in cALD compared with cAMN, with 5711 CpGs being more hypermethylated in children vs. 4186 CpGs more hypermethylated in adults. The difference was even higher when we considered only CpGs in the more extreme ranges; for the β‐values above 0.1, we found 872 CpGs in cALD vs. 411 in cAMN (*P* = 2.4 E – 38), and for the β‐values below −0.1, we found 620 in cALD vs. 377 in cAMN (*P* = 1.2 E – 18) (Figure 5C). Interestingly, we also detected a higher mean hypermethylation of CpGs in the set of genes differentially methylated between cALD and cAMN, that are oligodendrocyte‐specific (Figure 5F), and also in the set of genes that are downregulated during differentiation of oligodendroglial precursor cells (Figure 5I). We also find more genes overlapping with a gene set harboring H3K27me3 marks in their promoters in cALD compared with cAMN (Figure 5L; see genes associated to pathways in Supporting Information Table S2). Regarding individual genes belonging to these pathways, in the cALD phenotype we found significantly increased hypermethylation of the CpGs of genes such as *CNP* (*P* = 5.4 E – 03), *MBP* (*P* = 0.048), *NINJ2* (*P* = 0.021), SRY‐box 10, *SOX10,* (*P* = 0.02) and NHS like 1, *NHSL1,* (*P* = 3.54 E – 05), all involved in oligodendrocyte differentiation. Other genes with H3K27me3 marks in their promoters showed also increased hypermethylation in the cALD phenotype, such as homeobox A3, *HOXA3,* (*P* = 5.46 E – 13) and ArfGAP with GTPase domain, ankyrin repeat and PH domain 2, *AGAP2,* (*P* = 7.95 E – 06) thus correlating a more marked degree of methylation with a more severe phenotype.

### Validation and integrative analysis of methylomic and transcriptomic data

**Figure 6:**
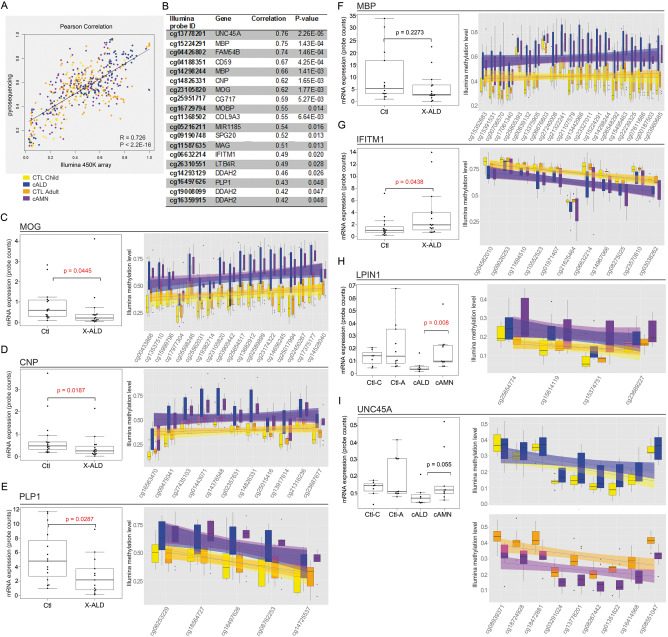
*Concordant methylation changes with gene expression*. Pearson's correlation between methylation measured by the Illumina array and pyrosequencing assays. Plot shows methylation data in 30 DNA brain samples in 22 different CpGs (**A**). Summary of Pearson's correlations of CpG methylation at sites measured by both Illumina array and pyrosequencing assays (**B**). Box‐and‐whisker plot of quantitative PCR normalized log2 expression in brain samples concordant with differential methylated CpGs within a DMR in associated genes (**C**–**I**). Concordant hypermethylation and downregulated expression in the oligodendrocyte markers *MOG*, *CNP*, *PLP1* and *MBP* (C–F), hypomethylated DMR and upregulation of the immune‐related gene *IFITM1* (G), hypermethylation in X‐ALD and differential expression between cALD and cAMN for *LPIN1* (H) and concordant methylation‐expression levels with differential expression between cALD and cAMN for *UNC45A* (I). Methylation levels for X‐ALD and controls are plotted in the box plot, with lines connecting each consecutive CpG assayed fitting a linear model. Whiskers indicate 1.5 times the interquartile range; bottom and top of the boxes, first and third quartiles, respectively; center lines, second quartile. Significant differences were determined by Student's *t*‐test or Wilcoxon rank sum test, and Kruskall–Wallis or Anova for more than two levels, according to the Shapiro–Wilk normality test.

To verify that the changes in methylation detected by the Illumina array were reliable and accurate, we used an independent approach using pyrosequencing. This technique quantifies methylated and unmethylated cytosines on the basis of the detection of pyrophosphate release on nucleotide incorporation after bisulfite conversion. We selected 22 CpGs within DMRs of interest and performed pyrosequencing analysis of 30 brain prefrontal cortex areas. We obtained a significant Pearson correlation of 0.725 with *P* < 2.2 E – 16 between the Illumina array and the pyrosequencing assay in the global analysis of 482 CpGs (Figure 6A,B), thus indicating a good association between the two sets of data. Furthermore, we performed quantitative PCR from brain samples of X‐ALD patients and controls to investigate whether the changes in the methylome translated into gene expression. Indeed, we detected concordant changes between hypermethylation and downregulated expression for the myelin genes *MOG, CNP*, *PLP1* and *MBP* (Figure 6C–F) in both childhood and adult X‐ALD samples. For *MBP*, the difference in expression showed a trend that reflected the methylation pattern (ie, hypermethylation and downregulated expression in X‐ALD samples), although without reaching significance, probably owing to the reduced sample size (Figure 6F). For *IFITM1*, interferon‐induced transmembrane protein 1, in contrast, we detected a hypomethylation and concordant upregulated expression in both phenotypes (Figure 6G). A similar pattern was observed for the enzyme lipin 1 (LPIN1), a transcriptional coactivator with a key role in myelin formation and maintenance in Schwann cells (Figure 6H). Intriguingly, unc‐45 myosin chaperone A (*UNC45A*) showed differential methylation between cALD and cAMN samples, thus suggesting its potential use as a biomarker for discriminating children from adults with cerebral X‐ALD (Figure 6I). All of these DMRs were located within promoter regions with the exception of *CNP* and *LPIN1*, which were differentially methylated in the gene bodies. Notably, the DMRs in the *CNP* and *LPIN1* genes overlapped with DNase I‐hypersensitive areas, thus indicating putative regulatory regions 15.

**Figure 7:**
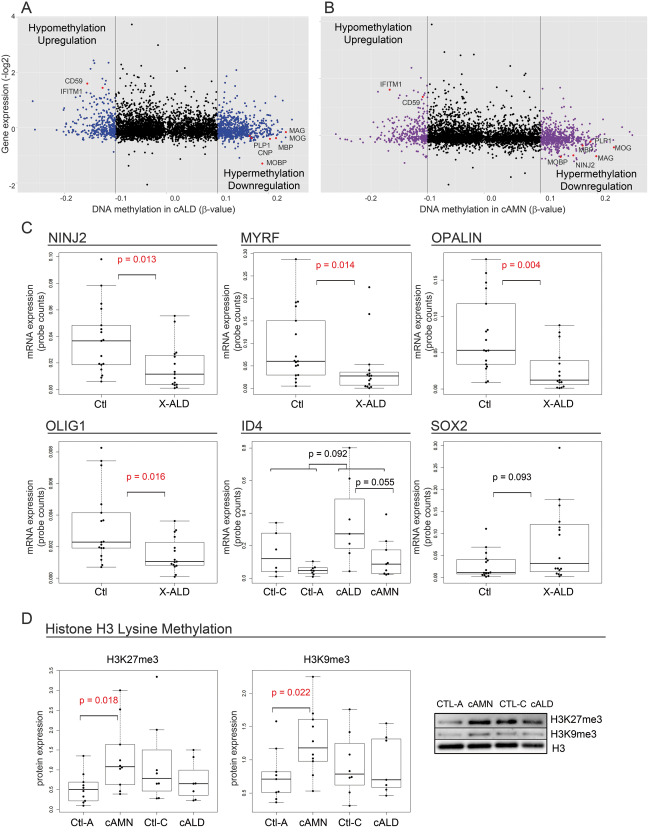
*Coordinated changes in DNA methylation and expression of associated genes for all overlapping genes between Illumina arrays and Affymetrix expression arrays*. On the *x*‐axis, the β‐value for the differentially methylated regions between X‐ALD patients and controls in the same age group for significant windows is shown; on the *y*‐axis, the log2 expression in cALD and cAMN samples (**A** and **B**) are shown, respectively. Box‐and‐whisker plot of quantitative PCR for *NINJ2*, *MYRF*, *OPALIN*, *OLIG1*, *ID4* and *SOX2* (**C**) and WB for H3K27me3 and H3K9me3 (**D**) in X‐ALD patients. Gene expression was normalized to that of the reference control gene human *RPLP0*. A representative immunoblot is shown. Protein levels are normalized relative to those of histone 3, and quantification is represented as intensities. Whiskers indicate 1.5 times the interquartile range; bottom and top of the boxes, first and third quartiles, respectively; center lines, second quartiles. Significant differences were determined by Student's *t*‐test or Wilcoxon rank sum test for pairwise comparisons, and Kruskall–Wallis or Anova for more than two levels, according to the Shapiro–Wilk normality test.

To gain a deeper understanding of the transcriptional consequences of the differential DNA methylation, we integrated our methylomic data set with an existing transcriptome experiment on prefrontal cortex non‐affected white matter from cALD and cAMN samples, using an Affymetrix Human Genome U133A expression array 62. We found a significant correlation between methylation and expression of the myelin and immune genes (Figure 7A,B). Among transcripts that were repressed in X‐ALD in both adults and children with respect to controls and hypermethylated gene‐associated DMRs, we detected downregulation of the myelin genes *CNP, MBP, PLP1, MOG, MOBP, MAG* and the ninjurin 2 protein (*NINJ2*), a cell surface adhesion protein that is upregulated in Schwann cells surrounding the distal segment of an injured nerve, promotes neurite outgrowth and is present in the enriched oligodendrocyte differentiation gene set (Supporting Information Table S5). We also detected the upregulation of hypomethylated genes belonging to immune‐related functions, such as *IFITM1* and *CD59*, a cell surface glycoprotein that regulates complement‐mediated cell lysis and is involved in lymphocyte signal transduction.

We next validated these results through an orthogonal approach using quantitative PCR of genes differentially expressed in the Affymetrix array and additional ones involved in oligodendrocyte maturation and differentiation (MYRF, OPALIN, OLIG1). Thus, in X‐ALD samples, we identified the significant downregulation of (i) *NINJ2*; (ii) the myelin regulatory factor *MYRF,* which is required for central nervous system myelination and oligodendrocyte differentiation; (iii) the oligodendrocytic myelin paranodal and inner loop protein *OPALIN*; (iv) and the oligodendrocyte transcription factor 1 *OLIG1*, which also appears to play a critical role in late‐stage oligodendrocyte maturation and myelin formation 56. OPCs are undifferentiated cells characteristically defined by high levels of transcriptional inhibitors, including the HLH family members HES1, HES5, ID2 and ID4. The expression of ID4 declines during oligodendrocyte differentiation into myelin‐forming cells and forced expression of either ID4 or ID2 prevents differentiation of OPCs *in vitro* 28. Subgroup B of the Sox family includes SOX1, 2 and 3, transcriptional activators, which are expressed in proliferating neural progenitors. Among these family members, SOX2 has been shown to increase proliferation, inhibit neuronal differentiation and support re‐programming into neural stem cells 36. SOX2 is downregulated during the late stages of oligodendrocyte maturation by HDAC activity 67. Because we detected differential levels of ID4 and SOX2 transcripts in Affymetrix expression arrays in X‐ALD patients compared with controls, we hypothesized that high *ID4* or *SOX2* levels may serve as an additional brake on the expression of myelin genes in X‐ALD brains. We thus validated these results by quantitative PCR and found a trend toward higher expression of *ID4* and *SOX2* in X‐ALD patients compared with controls, and a trend toward higher expression of ID4 in cALD than in cAMN (Figure 7C).

### Histone H3 lysine methylation

Our functional enrichment analysis also identified the hypermethylation of several genes with the repressive histone mark H3K27me3 in their promoters in both cALD and cAMN samples, compared with controls in the same age group. DNA methylation and histone modification, particularly methylation of residue K27 in H3, may be interdependent 10. We thus used western blotting to examine the levels of H3K27me3 protein in white matter from patients with cALD and cAMN and healthy male controls in the same age group and found an increase in the protein levels in the cAMN but not in the cALD samples (Figure 7D). A similar scenario was observed for H3K9me3, which we found at higher levels in cAMN but not in the cALD samples compared with controls in the same age group (Figure 7D). In line with these results, we found a stronger hypermethylation in cALD than in cAMN DNA promoters with the H3K27me3 mark. However, we did not observe a correlation between DNA and histone methylation in X‐ALD samples. A possible explanation for the lack of concordance between cALD and cAMN in DNA and histone methylation results may be the age difference between both phenotypes. The post‐mitotic stage of cells in adult cAMN may favor a definitive terminal differentiation induced by H3K27 and H3K9 lysine methylation. In contrast, the plasticity in younger cALD cells may allow for a regulation of gene expression by a more labile response, such as promoter DNA methylation.

### Identification of markers of disease onset

**Figure 8:**
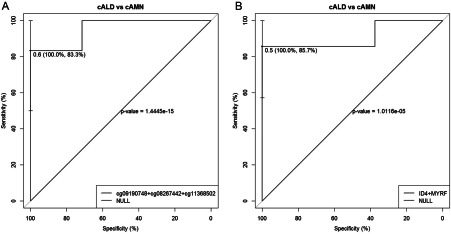
*Penalized logistic regression models in X‐ALD*. Best ROC curves for penalized logistic regression models using methylation data from 22 CpGs in 23 brain samples. In (**A**), the combination of the CpGs cg09190748 (*SPG20* promoter), cg08267442 (*UNC45A* promoter) and cg11368502 (*COL9A3* promoter) discriminate 100% of the cALD cases and 83.3% of the cAMN cases (AUC = 0.95). In (**B**), the gene expression of *ID4* and *MYRF* discriminate 100% of the cALD cases and 85.7% of the cAMN cases (AUC = 0.91). The DeLong test was used to compare the areas under two different correlated ROC curves.

To identify the best disease markers capable of discriminating between cALD and cAMN, we used the methylation data of the 22 CpGs tested to build a penalized logistic regression model from the pyrosequencing values of 23 white matter samples (6 cALD, 7 cAMN, 3 child controls and 7 adult controls). To ensure robustness of the penalized regression model, the analysis included a leave‐one‐out cross‐validation (LOOCV) procedure characterized by a single sample as validation data for testing the model while the remaining samples were used as training data (see Materials and Methods). The cross‐validation process was then repeated as many times as the number of samples used in the model. The model results are presented as the Area Under the Curve (AUC) of the Receiver Operator Characteristic (ROC). The best model obtained was the combination of the CpGs cg09190748 (*SPG20* promoter), cg08267442 (*UNC45A* promoter) and cg11368502 (*COL9A3* promoter) (Figure 8A). This model produced an area under the curve (AUC) of 0.93 (LOOCV) and a significant DeLong's test for two correlated ROC curves with a *P* value of 1.44 E – 15 against the reduced hypothesis that the AUC was 0.5, which would indicate that a model does not discriminate between phenotypes. With this data set, the model has a true positive rate or sensitivity of 100% and a true negative rate or specificity of 83.3%. The sensitivity measures the proportion of one of the X‐ALD phenotypes that is correctly identified as such (eg, the percentage of cALD patients who are correctly identified as having the condition), and specificity measures the proportion of the other X‐ALD phenotype that is correctly identified as such (eg, the percentage of cAMN patients who are correctly identified as cAMN). This result indicated that cALD and cAMN phenotypes can be differentiated using the combined CpG methylation values of the *SPG20, UNC45A* and *COL9A3* promoters.

Following a similar approach, we searched for the best model capable of discriminating between childhood and adult X‐ALD, using expression data from the reported quantitative PCR results (Figure 8B). We assessed the suitability of 13 different genes related to oligodendrocyte differentiation and immune response. We found that the model with the combined expression data of *ID4* and *MYRF* had an ROC area of 0.91 and a *P* value of 1.01 E – 05 for the null hypothesis that the ROC curve area was 0.5. The model has a true positive rate or sensitivity of 100% and a true negative rate or specificity of 85.7%, thus indicating that the combined mRNA expression values of *ID4* and *MYRF* can discriminate cALD from cAMN samples.

## Discussion

A main conclusion of the current study is that differential DNA methylation of genes affecting oligodendrocyte differentiation and myelin formation may provide an explanation for the hampered remyelination observed in X‐ALD patients. Oligodendrocytes produce myelin sheaths that wrap around neuronal axons in the central nervous system, thereby facilitating rapid signal transmission. They are very susceptible to ischemic or oxidative damage, thus leading to demyelination 3. In X‐ALD, the inflammatory phenotypes are characterized by cerebral demyelination with the death of oligodendrocytes 52, 68.

Loss of myelin may be counteracted by the formation of new myelin by resident oligodendrocyte progenitors, which are spread out throughout the brain parenchyma and have the ability to differentiate into myelinating oligodendrocytes 23, 77. Successful differentiation of progenitors into oligodendrocytes results from the integration of transcriptional networks with epigenetic modifications 43 including post‐translational modifications of lysine residues on nucleosomal histones 42, 47, 65 and changes in DNA methylation 54. New myelin synthesis is preceded by downregulation of oligodendrocyte differentiation inhibitors 41 and other molecules characteristic of the progenitor state. This is a consequence of the recruitment of histone deacetylases (HDACs) and repressive histone methyl transferases, thus allowing the temporal downregulation of inhibitors followed by increased transcription of myelin genes 28, 73. The transcriptional response associated with remyelination is characterized by decreased levels of *SOX2* and other transcriptional inhibitors such as *HES1, HES5, ID2* and *ID4*, followed by increased transcription of myelin genes 67. Here, we demonstrated that distinct clinical phenotypes of X‐ALD are associated with increased levels of transcriptional inhibitors of myelin gene expression (ie, I*D4* and S*OX2*) and the resulting decreased levels of genes associated with the mature differentiated phenotype (ie, *MBP, MOBP, MOG, MAG, NINJ2, OPALIN, OLIG1, CNP* and *PLP1*). This conclusion is consistent with the impaired remyelination capacities of oligodendrocytes in cases of adrenoleukodystrophy with distinctive clinical disease progression and with the neuropathological evidence supporting a lack of remyelination. A high turnover of the oligodendrocytic lineage, with early death of mature oligodendrocytes and an increased compensatory proliferation of the OPCs is also compatible with this result. Recently, a whole‐genome transcriptome and methylome analysis comparing OPCs vs. myelinating oligodendrocytes has revealed that DNA methylation is inversely correlated with gene expression during developmental myelination 54. In that work, the authors found that *MOG* and *MAG* were hypomethylated and upregulated during oligodendrocyte differentiation, whereas in X‐ALD, we found these genes hypermethylated and downregulated. Together, our data predict a sustained, compromised function of myelinating oligodendrocytes in X‐ALD.

Remarkably, we found ID4 (*P* = 0.055) more expressed in cALD compared with cAMN, suggesting a more marked repression of myelin genes in the most severe phenotype. Along the same lines, and albeit both phenotypes show hypermethylation of CpGs in promoters, we found higher levels of hypermethylation in oligodendrocyte‐differentiating and myelin genes such as *MBP*, *SOX10, CNP* and *NINJ2,* in cALD compared with cAMN. Decreased remyelination efficiency and impaired OPC differentiation characterize aging, together with a progressive loss of epigenetic memory in mature oligodendrocytes 66, 67. Inefficient OPC differentiation in aging mirrors non‐remyelinating plaques in humans with multiple sclerosis, which are replete with oligodendrocyte‐lineage cells that fail to differentiate into remyelinating oligodendrocytes 76. In demyelinated brains in elderly individuals, HDAC recruitment is inefficient, thus allowing for the accumulation of transcriptional inhibitors and preventing the subsequent surge in myelin gene expression. Redox status, mitochondrial fitness and proteostasis capacities are affected during and may contribute to aging 46. In X‐ALD, we have recently uncovered an early‐onset oxidative damage caused by excess VLCFA that, together with mitochondria malfunction and impairment of proteasome and autophagy activation, may be a major contributor to disease pathogenesis 18, 19, 20, 38, 39, 44, 45, 51. We thus hypothesize that an “accelerated aging scenario” may affect the epigenetic regulation of myelin regeneration as an epimutagenic contributor to the pathogenesis of X‐ALD. However, in light of recent findings linking enriched environment to oligodendrocyte differentiation 34, 78, we cannot rule out that a deprived environment related to disease progression exerts a detrimental effect in the differentiation process.

Pathological‐appearing axons in multiple sclerosis lesions are associated with neuroinflammation, that is, the presence of activated microglia/macrophages, astrocytes and lymphocytes 16, 57, which are considered to be a driving force of demyelination and axonopathy 6. Notably, the order of events leading to myelin‐related pathology, such as redundant myelin profiles, demyelination, axonopathy, neuroinflammation and lymphocyte recruitment, differs between distinct models and diseases 55. In X‐ALD, it seems plausible that the development of the severe cerebral phenotypes may be enhanced by immune responses that target oligodendrocytes and abnormal myelin with excess VLCFA, thus resulting in demyelination and reactive gliosis 17, 31. However, in contrast to that of multiple sclerosis, the pathogenesis in cerebral X‐ALD, although insufficiently characterized, does not appear to be determined by a T‐cell–driven conversion of an innate to adaptive immune response. Instead, microglial apoptosis in perilesional white matter may represent an early stage in lesion evolution 14. Although the oligodendrocytes and axons are the evident targets in cerebral X‐ALD, the loss of microglia and/or abnormal microglia function may impair the ability to provide neuroprotective factors to deficient oligodendrocytes. Injury to oligodendrocytes may be enhanced via an inflammatory response that follows tissue injury and plays an important role by initiating and accelerating the progression of the disease.

Distinct or global changes in the epigenetic landscape are hallmarks of chronic inflammation‐driven diseases. There is ample evidence suggesting that epigenetic mechanisms may mediate the development of chronic inflammation by modulating the expression of proinflammatory cytokines such as TNF and interleukins and the autocrine and paracrine activation of the transcription factor NF‐kB 63, which we have recently described as being activated in the spinal cord of the mouse model of X‐ALD 62 and peripheral blood of pure AMN patient samples 59.

Our present findings in X‐ALD identified the differential methylation in the DMRs of the immune‐associated genes *IFITM1* and *CD59*. Interestingly, the *IFITM1* gene, which is overexpressed in X‐ALD, belongs to the interferon signature pathway and plays a role in the host defense against intracellular bacterial/viral infection. This gene has previously been reported to be hypomethylated in autoimmune diseases such as primary Sjögren's syndrome 1 and inflammatory bowel disease 27. We also identified hypermethylation of *LPIN1* in both X‐ALD phenotypes, with a lower gene expression in childhood cALD compared with late‐onset cAMN. This lipid metabolic enzyme has been found to be involved in macrophage signaling and animal responses to bacterial components as a proinflammatory mediator during TLR signaling 49. Of note, the *UNC45A* gene exhibits an intriguing pattern with hypermethylation and repressed expression in cALD samples, as compared with hypomethylation and overexpression in cAMN. UNC45A is a highly conserved member of the UNC45/CRO1/She4p family of proteins, which acts as chaperones for both conventional and nonconventional myosin involved in cytokinesis, cell motility and organelle trafficking. Specifically, UNC45A knockdown causes impaired NK‐mediated cytotoxicity by preventing lytic granule exocytosis and, thus, decreasing IFN‐γ secretion 33. This pathway is of interest because a CNS‐specific deficit of NK cellular functions may lead to aggressive inflammation and autoimmunity. Indeed, during EAE in C57BL/6 mice, depletion of NK cells exacerbates neurological deficits 26, 30. We thus propose that decreased UNC45A function may contribute to exacerbated, earlier onset neuroinflammation in children with cALD.

**Figure 9:**
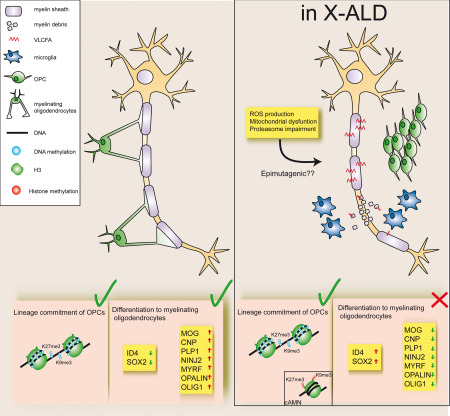
*Working model of the impaired remyelination process in X‐ALD*. VLCFA accumulation in X‐ALD changes the membrane lipid composition in the ganglioside, phosphatidylcholine, proteolipid and cholesterol ester fractions of brain myelin. In response to this demyelinating insult, microglial cells are recruited to the demyelinated area, where they release mediators that mobilize oligodendrocyte precursor cells (OPCs). These precursors are continuously generated from neural stem cells, to the detriment of neuronal precursor cells, and further differentiate into mature myelinating oligodendrocytes and form a thinner myelin sheath around the demyelinated axon. In X‐ALD, however, the oligodendrocyte‐lineage cells fail to differentiate into remyelinating oligodendrocytes. There is a lineage commitment of OPCs in X‐ALD characterized by DNA methylation in genes implicated in the restriction of multipotentiality and heterochromatin formation with histone marks H3K9me3 and H3K27me3 at their promoters (blue circles) and histone modifications H3K9me3 and H3K27me3 of histone H3 (red circles), particularly in cAMN. Nevertheless, the downregulation of oligodendrocyte differentiation inhibitors such as *ID4* and *SOX2*, followed by increased transcript levels of myelin genes such as *OLIG1*, *MOG*, *CNP*, *PLP1*, *MYRF*, *OPALIN* and *NINJ2* preceding the differentiation of myelinating oligodendrocytes, is impaired in X‐ALD.

In conclusion, we suggest that aberrant epigenetic regulation may account for the impairment in the remyelinating capabilities of oligodendrocytes and the activation of the immune response in X‐ALD, more severe in children with cALD, as shown in the working model in Figure 9. We propose four different epigenetic mechanisms operating in the X‐ALD inflammatory brains: (i) a repressive methylation at promoters of genes with the H3K27me3 mark and histone modification of lysine residues K9 and K27 on histone H3, especially in cAMN; (ii) the hypermethylation of promoters and the ensuing repression of myelin genes; (iii) the upregulated expression of the transcriptional inhibitors *ID4* and *SOX2*, thus suggesting aberrant activity of HDACs; (iv) and differential methylation in immune‐associated genes such as *LPIN1*, *IFITM1* and *UNC45A*. The transcriptional and translational evidences suggest that inefficient oligodendrocyte differentiation may occur as a consequence of a perturbed epigenetic regulation of gene expression, thus leading to a dysregulated transcriptional response in X‐ALD. These alterations may create a cellular context characterized by altered transcriptional programs of OPC differentiation, which may impair the maintenance of myelin sheaths, thereby preventing the appropriate remyelination of X‐ALD brain lesions.

The identification of methylomic and transcriptomic markers that discriminate between the childhood and adult onset of cerebral disease is a first step toward the characterization of markers of prognostic value that may help inform therapeutic choices. These results warrant validation in different cohorts. Additionally, inclusion of pure AMN white matter samples from patients without inflammatory demyelination and from asymptomatic ABCD1 mutation carriers, both too rare to be included in the experimental setup of this current work, will be necessary to complete a comprehensive picture of disease states.

## Conflict of Interest

None declared

## Web Resources

Molecular Signatures Database (MSigDB) data set: http://software.broadinstitute.org/gsea/index.jsp

RGL package: https://r-forge.r-project.org/projects/rgl/.

## Supporting information


**Table S1.** List of samples analyzed. White matter tissues from cALD, cAMN patients and healthy age‐matched male control subjects.

**Table S2.** Functional enrichment in hypermethylated DMR‐associated genes in X‐ALD brains. Molecular Signatures Database (MSigDB) data set enrichment in hypermethylated DMR‐associated genes in X‐ALD with respect to controls by computing a hypergeometric distribution with Benjamini–Hochberg Multiple Testing Correction. ID, MSigDB identification; Description, MSigDB gene set description; Adjusted *P* value, *P* values for each gene set tested adjusted by fdr; count, number of genes differentially methylated that are annotated at the gene set; size, number of genes from the 450K array that are annotated at the gene set.

**Table S3.** Functional enrichment in hypomethylated DMR‐associated genes in X‐ALD brains. Molecular Signatures Database (MSigDB) data set enrichment in hypomethylated DMR‐associated genes in X‐ALD with respect to controls by computing a hypergeometric distribution with Benjamini–Hochberg Multiple Testing Correction. ID, MSigDB identification; Description, MSigDB gene set description; Adjusted *P* value, *P* values for each gene set tested adjusted by fdr; count, number of genes differentially methylated that are annotated at the gene set; size, number of genes from the 450K array that are annotated at the gene set.

**Table S4.** Functional enrichment in DMR‐associated genes in X‐ALD children with respect to those in X‐ALD adults. Molecular Signatures Database (MSigDB) data set enrichment in DMR‐associated genes in X‐ALD children with respect to those in X‐ALD adults by computing a hypergeometric distribution with Benjamini–Hochberg Multiple Testing Correction. ID, MSigDB identification; Description, MSigDB gene set description; Adjusted *P* value, *P* values for each gene set tested adjusted by fdr; count, number of genes differentially methylated that are annotated at the gene set; size, number of genes from the 450K array that are annotated at the gene set.

**Table S5.** Enrichment of the gene set: (i) methylated genes in OPC to OL transition and (ii) genes with H3K9me3 and H3K27me3 marks in OPCs and differentiating OLs; in XALD differentially methylated DMRs associated genes. Pathway, gene set definition; count, number of genes differentially methylated that are annotated at the gene set; size, number of genes from the 450 k Array that are annotated at the gene set, *P* value, *P* values for each gene set tested by Fisher's exact test; genes associated, genes differentially methylated in XALD that overlap with the given gene set.
