# Supplementary material for: Epigenomic signature of adrenoleukodystrophy predicts compromised oligodendrocyte differentiation
Source: Brain Pathol. 2018 Apr 10;28(6):902–19. doi: 10.1111/bpa.12595 (PMC6857458; doi:10.1111/bpa.12595)
Supplement: Supplementary file 1 — Table S1. List of samples analyzed. White matter tissues from cALD, cAMN patients and healthy age‐matched male control subjects. [file BPA-28-902-s001.docx]

| ID | Cerebrum | Disorder | Age | Sexe | Race |
| --- | --- | --- | --- | --- | --- |
| cALD1 | Prefrontal cortex | cALD | 6 | Male | Caucasian |
| cALD2 | Prefrontal cortex | cALD | 8 | Male | Caucasian |
| cALD3 | Prefrontal cortex | cALD | 8 | Male | Caucasian |
| cALD4 | Prefrontal cortex | cALD | 9 | Male | Caucasian |
| cALD5 | Prefrontal cortex | cALD | 9 | Male | Caucasian |
| cALD6 | Prefrontal cortex | cALD | 10 | Male | Caucasian |
| cALD7 | Prefrontal cortex | cALD | 13 | Male | Caucasian |
| cALD8 | Prefrontal cortex | cALD | 13 | Male | Caucasian |
| cAMN1 | Prefrontal cortex | cAMN | 35 | Male | Caucasian |
| cAMN2 | Prefrontal cortex | cAMN | 39 | Male | Caucasian |
| cAMN3 | Prefrontal cortex | cAMN | 43 | Male | Caucasian |
| cAMN4 | Prefrontal cortex | cAMN | 44 | Male | Caucasian |
| cAMN5 | Prefrontal cortex | cAMN | 47 | Male | Caucasian |
| cAMN6 | Prefrontal cortex | cAMN | 48 | Male | Caucasian |
| cAMN7 | Prefrontal cortex | cAMN | 58 | Male | Caucasian |
| cAMN8 | Prefrontal cortex | cAMN | 63 | Male | Caucasian |
| cAMN9 | Prefrontal cortex | cAMN | 68 | Male | Caucasian |
| CA1 | Prefrontal cortex | Control Adult | 37 | Male | Caucasian |
| CA2 | Prefrontal cortex | Control Adult | 38 | Male | Caucasian |
| CA3 | Prefrontal cortex | Control Adult | 47 | Male | Caucasian |
| CA4 | Prefrontal cortex | Control Adult | 57 | Male | Caucasian |
| CA5 | Prefrontal cortex | Control Adult | 57 | Male | Caucasian |
| CA6 | Prefrontal cortex | Control Adult | 57 | Male | Caucasian |
| CA7 | Prefrontal cortex | Control Adult | 58 | Male | Caucasian |
| CA8 | Prefrontal cortex | Control Adult | 62 | Male | Caucasian |
| CA9 | Prefrontal cortex | Control Adult | 64 | Male | Caucasian |
| CC1 | Prefrontal cortex | Control Child | 4.5 | Male | Caucasian |
| CC2 | Prefrontal cortex | Control Child | 4.5 | Male | Caucasian |
| CC3 | Prefrontal cortex | Control Child | 5 | Male | Caucasian |
| CC4 | Prefrontal cortex | Control Child | 7 | Male | Caucasian |
| CC5 | Prefrontal cortex | Control Child | 8 | Male | Caucasian |
| CC6 | Prefrontal cortex | Control Child | 11 | Male | Caucasian |
| CC7 | Prefrontal cortex | Control Child | 12 | Male | Caucasian |
| CC8 | Prefrontal cortex | Control Child | 13 | Male | Caucasian |

**Additional file 1:Table S1**: Supplementary Table 1: List of samples analyzed. White matter tissues from cALD, cAMN patients and healthy age-matched male control subjects.
