# Supplementary material for: Epigenomic signature of adrenoleukodystrophy predicts compromised oligodendrocyte differentiation
Source: Brain Pathol. 2018 Apr 10;28(6):902–19. doi: 10.1111/bpa.12595 (PMC6857458; doi:10.1111/bpa.12595)
Supplement: Supplementary file 2 — Table S2. Functional enrichment in hypermethylated DMR‐associated genes in X‐ALD brains. Molecular Signatures Database (MSigDB) data set enrichment in hypermethylated DMR‐associated genes in X‐ALD with respect to controls by computing a hypergeometric distribution with Benjamini–Hochberg Multiple Testing Correction. ID, MSigDB identification; Description, MSigDB gene set description; Adjusted P value, P values for each gene set tested adjusted by fdr; count, number of genes differentially methylated that are annotated at the gene set; size, number of genes from the 450K array that are annotated at the gene set. [file BPA-28-902-s002.docx]

| Pathway | **count** | **size** | **Adjusted P-value** | **DMR genes associated** |
| --- | --- | --- | --- | --- |
| **OLIGODENDROCYTE MARKERS** | 18 | 71 | 1.57E-11 | CNTN2/MOBP/MOG/SOX10/GJB1/AATK/SCD/MBP/PTGDS/GSN/PACS2/MAG/CNP/C11orf9/ABCA2/SGK1/OPALIN/TMEM88B/ |
| **ES WITH H3K27ME3** | 65 | 1094 | 1.25E-08 | CALCA/SCD5/TMEFF2/FGF20/DUSP6/MICB/FAM123A/PAX3/MAB21L2/GATA6/ESR1/EXOC3L2/RASGEF1C/AGAP2/HOXA3/TFAP2B/MSC/CNTN2/C4orf49/HHIP/RPS6KA2/ECEL1/NFIX/CA10/GPM6B/OSR2/LRBA/GSN/CBX4/HOXB7/ALX4/PRDM13/HOXA2/PTPRN2/FOXD2/ARID5B/EBF1/ARL5C/TWIST1/FEZ1/SOX8/CCDC140/EBF3/FLI1/HOXB1/GDF7/RGS10/PRRT1/COL9A2/CHN2/HOXB3/HOXB6/SYNE1/WNT6/DIO3/HOXA6/CNNM2/HOXA4/SKAP1/DLX5/LHX6/CACNB4/GRID1/HS3ST3B1/NKX6-1/ |
| **OLIGODENDROCYTE DIFFERENTIATION DN** | 58 | 1041 | 1.39E-06 | DBNDD2/BRI3BP/PDLIM2/BAZ2B/SH3BP4/ADD3/HIP1R/PTPRE/MBP/TRIM2/FOXP2/GPD1/DSCAM/NAV1/MARCKS/KALRN/SOX8/PTGDS/SMURF1/TMCC3/PACS2/TBC1D16/CYTH1/BRSK1/TMEFF2/ANK3/TMEM88B/FAM53B/SASH1/AATK/CNP/NINJ2/LSAMP/MYO1G/SYNE1/SLC25A10/SLC15A2/RBMS1/TULP4/FGFR2/THSD7A/GNG7/ARHGAP23/ADO/C11orf9/DDR1/CHN2/COL11A2/SNED1/TJAP1/NDRG4/KIAA1522/ABCA2/VPS37B/C9orf3/SGK2/NHSL1/SYNPO/ |
| **MAJOR DEPRESSIVE DISORDER DN** | 18 | 156 | 8.38E-06 | SOX10/ST18/CNTN2/CNP/P2RX7/BCAT1/SYNJ2/FGFR2/LDB3/MAG/RNASE1/ABCA2/FAM53B/C11orf9/MOG/PIK3R1/SORBS2/MOBP/ |
| **MEF HCP WITH H3K27ME3** | 37 | 576 | 2.07E-05 | PRRT3/SKAP1/CNTN2/PAQR6/SALL4/HOXB3/FGF20/PRSS50/DSCAM/CA10/PRDM8/PHACTR1/HOXB1/SCUBE1/EXOC3L2/TFAP2B/ALX1/PFN3/PTPRN2/RAP1GAP2/TTC22/KIF12/MCF2L/RASGEF1C/DLEU7/KIAA1598/PRRT1/RASAL1/ECEL1/TACR3/GRID1/ACCN4/PRKCZ/SCGN/TNXB/AATK/NKX6-1/ |
| **BRAIN HCP WITH H3K27ME3** | 23 | 264 | 2.16E-05 | MARCKSL1/GDF7/HOXB6/DIO3/SALL4/ALX1/TNXB/FOXD2/HOXB9/ECEL1/FGF20/ALX4/PRSS50/C1orf187/PRDM13/HOXA4/WDR86/HOXB7/NKX6-1/HOXA5/ATP2A3/TNFAIP2/HOXB4/ |
| **BREAST CANCER 7P15 AMPLICON** | 5 | 11 | 5.10E-05 | HOXA4/HOXA2/HOXA3/HOXA5/HOXA6/ |
| **STEM CELL DN** | 19 | 212 | 0.00012757 | DUSP6/UPP1/GPR56/KIF25/SEC14L1/HOXB7/SCARB1/THSD7A/HLA-DPB1/MCF2L/CYTH1/RPS6KA2/FLI1/RAPGEF5/RNASE1/NOTCH4/PTPRE/ARHGEF15/LHX6/ |
| **SOX9 TARGETS IN PROSTATE DEVELOPMENT UP** | 6 | 20 | 0.00013367 | MBP/HOXB9/LHX6/TRIM2/FGFR2/DUSP6/ |
| **EED TARGETS** | 51 | 1036 | 0.00021388 | FEZ1/FGF20/CCDC140/FOXD2/DLX5/RIN3/GATA6/FLI1/DIO3/PAX3/HOXA2/MLNR/GDF7/HOXB6/GRID1/HOXB7/TWIST1/HOXB3/C4orf49/PRDM13/EBF1/COL9A2/MAB21L2/ALX4/HOXA3/TMEFF2/MRPS18B/TFAP2B/ALX1/CALCA/MICB/WNT6/CHN2/MSC/MTSS1/SCD5/HOXB1/HHIP/HOXA6/CNNM2/HOXA4/NFIX/ECEL1/LHX6/HIST1H3E/HS3ST3B1/SLC8A3/CA10/RGS10/GPM6B/NKX6-1/ |

**Additional file 2: Table S2**. Functional enrichment in hypermethylated DMR-associated genes in X-ALD brains. Molecular Signatures Database (MSigDB) data set enrichment in hypermethylated DMR-associated genes in X-ALD with respect to controls by computing a hypergeometric distribution with Benjamini–Hochberg Multiple Testing Correction. ID, MSigDB identification; Description, MSigDB gene set description; Adjusted p-value, p values for each gene set tested adjusted by fdr; count, number of genes differentially methylated that are annotated at the gene set; size, number of genes from the 450K array that are annotated at the gene set.
