# Supplementary material for: Epigenomic signature of adrenoleukodystrophy predicts compromised oligodendrocyte differentiation
Source: Brain Pathol. 2018 Apr 10;28(6):902–19. doi: 10.1111/bpa.12595 (PMC6857458; doi:10.1111/bpa.12595)
Supplement: Supplementary file 3 — Table S3. Functional enrichment in hypomethylated DMR‐associated genes in X‐ALD brains. Molecular Signatures Database (MSigDB) data set enrichment in hypomethylated DMR‐associated genes in X‐ALD with respect to controls by computing a hypergeometric distribution with Benjamini–Hochberg Multiple Testing Correction. ID, MSigDB identification; Description, MSigDB gene set description; Adjusted P value, P values for each gene set tested adjusted by fdr; count, number of genes differentially methylated that are annotated at the gene set; size, number of genes from the 450K array that are annotated at the gene set. [file BPA-28-902-s003.docx]

| Pathway | **count** | **size** | **Adjusted P-value** | **DMR genes associated** |
| --- | --- | --- | --- | --- |
| **BOUND BY PML RARA FUSION** | 49 | 449 | 4.42E-12 | KCNAB2/FOSL2/FAM129C/TOX2/SKI/RASA3/DEF6/SMAD3/GAS7/CACNA1C/TMC8/RPS6KA2/BANP/PLCB2/MX2/MIR23A/ACP5/SLC9A3/DNM2/RUNX1/HPCAL1/ZBTB16/TPPP3/PLEKHA2/RUNX3/CUX1/NFE2/PLEK/HMGA1/AJAP1/PLEKHO1/SPN/SNX20/C11orf21/LCP2/ABI3/TNFRSF4/SLC9A1/LFNG/MAP2K3/PTPN6/LTB4R/PITPNM2/RAP1GAP2/FERMT3/RAB11FIP1/LASP1/ACSF3/MIR24-2/ |
| **PROSTATE CANCER RACE UP** | 34 | 282 | 2.19E-09 | LCP2/RUNX3/POU2AF1/MX1/OAS2/GLRX/XAF1/ICAM2/PSMB8/APOL1/GMFG/SLFN12/IFI27/ZAP70/ARHGAP25/PIM2/GNG11/IFITM1/CD93/SECTM1/SLAMF1/PSMB9/PRF1/PLEK/EDNRA/LSP1/TAP1/CD52/MX2/PTPN7/P2RY14/FHLA/HLA-F/HLA-E/ HLA-DPB1/ |
| **METABOLIC SYNDROME NETWORK** | 79 | 1185 | 8.20E-09 | NLRC3/ARHGDIB/HPCAL1/KCNN4/P2RY14/UNC13D/KLHL6/UNC93B1/THBS2/CD38/FAM198B/ABI3/GNMT/NIN/HVCN1/LCP2/FAM83A/PLEKHO1/TTC39C/FAM129A/CYFIP1/ITGBL1/TBC1D16/SPRED2/GPNMB/RASSF1/DOK2/LOX/HK3/RASA3/PHF11/FRMD4A/MAP3K8/C17orf62/VASN/PYCARD/PDLIM4/PTK2B/CAPN2/SPEG/SLC1A5/SP100/ABCG1/NLGN2/SNX20/PRR5L/NFAM1/RGS14/ADAMTS2/PLEK/TNFSF13B/CCRL2/CFP/INPP5D/GMFG/STAB1/PFKP/DNMT3A/GLRX/ANXA2/MPEG1/CD34/FERMT3/PTPN6/F10/LASP1/AIM2/HMGA1/ARHGAP25/RPS6KA2/HPGDS/LXN/FBLN2/MAN1C1/IL18BP/WDFY4/ARID3A/HLA-DMB/LGALS1/ |
| **BRAIN HCP WITH H3K4ME3 AND H3K27ME3** | 70 | 1058 | 1.43E-07 | CCDC88C/LHX2/PARP14/PRDM1/HLX/GNAS/TAP1/LRP5/CPNE5/WNT5A/FOXP4/EPB41L1/SLC9A3/CBLN1/HTRA4/GJB6/NR2E1/RAB11FIP1/MGAT5B/BCAR3/GDNF/SIX3/SIGIRR/FBLN2/PRDM16/ATP10A/NXN/SLC1A5/EMP2/KREMEN2/RUNX1/CASZ1/RAP1GAP2/HSPA2/HRH1/SLC16A3/NODAL/LTB4R2/INHBB/KIAA0146/FAM129A/ID3/PLEKHA2/SEMA5B/LFNG/PAX6/DMRTA2/SFRP1/PRR5L/LOX/GAD1/CMTM7/CDH22/WNT4/ADORA2A/ADAMTS2/OTX1/SHROOM3/SORCS2/SOX7/GPRC5C/ANXA2/WWTR1/VAX1/KCNH2/SFRP2/DHRS3/CHST8/ARX/ZIC5 |
| **TP53 TARGETS** | 71 | 1105 | 3.08E-07 | GDNF/TP73/MAN1C1/DIP2C/SMAD3/DLK1/APC2/MAN1A1/DEDD2/MCF2L/ZNF385A/ABCG1/COL9A3/LZTS1/KCNK3/MAD1L1/TOX2/DUSP13/GRAMD4/LTB4R/LRP5/VASN/ADAMTS2/SEPT9/SYNM/CAMTA1/KNDC1/EDN2/SORBS2/TTC39C/UNKL/LIMD1/MX1/ARX/ICAM2/GJB6/FAM198B/EDNRA/INPP5D/PRR5L/XAF1/ZCCHC14/HVCN1/ARHGEF4/GLB1L2/GNG11/GPRC5C/BLCAP/ACP5/KDM2B/ZAP70/GNAS/DHRS3/KLHL6/SH3TC1/C1orf168/CRIP2/GAD1/FOXP4/OBSCN/C1QTNF1/BGLAP/WNT7B/WIPI2/CBFA2T3/AFF1/MEGF6/WNT4/GNMT/GNG7/ HLA-DMB/ |
| **GATA2 TARGETS UP** | 21 | 146 | 3.08E-07 | FAM107B/NEURL3/IFITM1/LGALS1/KIAA0748/CERK/PAX6/ANXA2/PSMB9/CD93/GMFG/DGKG/PIK3CG/AFF1/MPO/CMTM7/PSMB8/P2RY14/KCNN4/ARID3A/ HLA-E/ |
| **INFLAMMATORY_RESPONSE LECTIN VS LPS DN** | 38 | 438 | 8.36E-07 | CD38/HRH1/SPN/AP2A2/UNC93B1/LCP2/STAB1/LXN/NAMPT/PARVG/LPCAT1/FRMD4A/KLF6/RBM47/HAMP/OAS2/MX1/C1S/TSPAN4/NT5C3/LIMD1/AIM2/NUPR1/GAS7/IFI27/C5orf13/XAF1/SP100/IFITM1/AFF1/PLEKHA2/MX2/GRAMD4/SECTM1/MAP3K8/GPNMB/NFAM1/ HLA-DMB/ |
| **BREAST CANCER 7P22 AMPLICON** | 10 | 34 | 1.30E-06 | GNA12/RADIL/FOXK1/C7orf50/LFNG/IQCE/WIPI2/CYP2W1/FAM20C/MAD1L1/ |
| **TARGETS OF RUNX1 RUNX1T1 FUSION HSC DN** | 22 | 183 | 2.79E-06 | NFE2/LTB/RAMP1/SLC43A3/DOK2/NOD2/ZBTB16/BIN2/KCNH2/S100A9/LGALS1/CBFA2T3/MGAT1/C11orf21/SLC16A3/RUNX3/STAB1/CD52/ARHGEF10/CST7/RGS14/LCP2/ |
| **AGING KIDNEY UP** | 38 | 463 | 2.79E-06 | PIK3CG/ANXA2/STAB1/HLA-DPA1/PLEKHO1/PPM1MGMFG/PARVG/MPEG1/ SHANK2/SYMPK/SPON2/LTB/ARHGDIB/LCP2/GPNMB/IFITM1/NR2F1/C17orf62/NRXN3/ARHGAP25/TAP1/FAM198B/MGAT1/PARP14/LPCAT1/C1S/PSMB8/RUNX3/PSMB9/PTPN6/MAP3K8/CD38/CMTM7/LASP1/ HLA-F/HLA-DMA/ HLA-E/ |

**Additional file 3: Table S3**. Functional enrichment in hypomethylated DMR-associated genes in X-ALD brains. Molecular Signatures Database (MSigDB) data set enrichment in hypomethylated DMR-associated genes in X-ALD with respect to controls by computing a hypergeometric distribution with Benjamini–Hochberg Multiple Testing Correction. ID, MSigDB identification; Description, MSigDB gene set description; Adjusted p-value, p values for each gene set tested adjusted by fdr; count, number of genes differentially methylated that are annotated at the gene set; size, number of genes from the 450K array that are annotated at the gene set.
