# Supplementary material for: Epigenomic signature of adrenoleukodystrophy predicts compromised oligodendrocyte differentiation
Source: Brain Pathol. 2018 Apr 10;28(6):902–19. doi: 10.1111/bpa.12595 (PMC6857458; doi:10.1111/bpa.12595)
Supplement: Supplementary file 4 — Table S4. Functional enrichment in DMR‐associated genes in X‐ALD children with respect to those in X‐ALD adults. Molecular Signatures Database (MSigDB) data set enrichment in DMR‐associated genes in X‐ALD children with respect to those in X‐ALD adults by computing a hypergeometric distribution with Benjamini–Hochberg Multiple Testing Correction. ID, MSigDB identification; Description, MSigDB gene set description; Adjusted P value, P values for each gene set tested adjusted by fdr; count, number of genes differentially methylated that are annotated at the gene set; size, number of genes from the 450K array that are annotated at the gene set. [file BPA-28-902-s004.docx]

| **Pathway** | **count** | **size** | **Adjusted P-value** | **DMR genes associated** |
| --- | --- | --- | --- | --- |
| **TYPE I DIABETES MELLITUS** | 3 | 41 | 0.00557968 | HLA-DOA/PTPRN2/HLA-E/ |
| **AUTOIMMUNE THYROID DISEASE** | 3 | 42 | 0.00557968 | HLA-DOA/TPO/HLA-E/ |
| **RESPONSE TO TZD DN** | 2 | 13 | 0.00557968 | CEBPD/CFD/ |
| **EICOSANOID LIGAND BINDING RECEPTORS** | 2 | 15 | 0.00663498 | LTB4R/LTB4R2/ |
| **IL22 SIGNALING UP** | 3 | 56 | 0.00903556 | SLFN12/PF4/CFD/ |
| **DOUBLE STRAND BREAK REPAIR** | 2 | 22 | 0.01479425 | LIG1/PRKDC/ |
| **PSMD4 TARGETS** | 3 | 71 | 0.01662539 | HLA-DQB2/HLA-E/HLA-DOA/ |

**Additional file 4: Table S4**. Functional enrichment in DMR-associated genes in X-ALD children with respect to those in X-ALD adults. Molecular Signatures Database (MSigDB) data set enrichment in DMR-associated genes in X-ALD children with respect to those in X-ALD adults by computing a hypergeometric distribution with Benjamini–Hochberg Multiple Testing Correction. ID, MSigDB identification; Description, MSigDB gene set description; Adjusted p-value, p values for each gene set tested adjusted by fdr; count, number of genes differentially methylated that are annotated at the gene set; size, number of genes from the 450K array that are annotated at the gene set.
