# Supplementary material for: Epigenomic signature of adrenoleukodystrophy predicts compromised oligodendrocyte differentiation
Source: Brain Pathol. 2018 Apr 10;28(6):902–19. doi: 10.1111/bpa.12595 (PMC6857458; doi:10.1111/bpa.12595)
Supplement: Supplementary file 5 — Table S5. Enrichment of the gene set: (i) methylated genes in OPC to OL transition and (ii) genes with H3K9me3 and H3K27me3 marks in OPCs and differentiating OLs; in XALD differentially methylated DMRs associated genes. Pathway, gene set definition; count, number of genes differentially methylated that are annotated at the gene set; size, number of genes from the 450 k Array that are annotated at the gene set, P value, P values for each gene set tested by Fisher's exact test; genes associated, genes differentially methylated in XALD that overlap with the given gene set. [file BPA-28-902-s005.docx]

| Pathway | count | size | P-value | genes associated |
| --- | --- | --- | --- | --- |
| Hypomethylated in OPCs to OL transition | 233 | 2214 | 5.33E-27 | MARCH10/SEPT8/AATK/ABCA2/ADAP1/ADARB2/ADD3/AFAP1/AGAP1/AGAP3/ALX4/ANK3/ANKRD11/ANKS1B/APC2/ARC/ARHGAP23/ARHGEF10/ARID3A/ARID5B/ARL5C/ASAP2/ATP11A/ATP2A3/ATP6V0A4/ATXN1/BAI1/BAZ2B/BCAR3/BCAT1/BCL11B/BRD4/BTBD17/CACNA1C/CACNA2D4/CACNB4/CAMTA1/CAPN2/CARD11/CASKIN2/CCRL2/CD6/CDH9/CHN2/CHST8/CIT/CMIP/CNP/CNTN1/COL11A2/COL5A1/COMT/CORO1B/CREB5/CUX1/CUX2/CXXC5/CYTH1/DBNDD2/DEF6/DENND1C/DEPDC5/DHCR24/DNM2/DNMT3A/DPYSL2/DSCAM/EFR3B/EHD1/EMP2/EP400/ERI3/ESRRG/FAM101A/FAM53B/FAM83A/FARP1/FBXL18/FBXO21/FBXW8/FERMT3/FGFR2/FOXK1/GALNT2/GAS7/GJC2/GNA12/GNAS/GNG7/GPD1/GPR37L1/GPR56/GPSM3/GRAMD1B/GRAMD4/GRAP2/GRID1/GRIK4/GRIN2B/GRM5/GSN/HIP1R/HIVEP3/HPCAL1/HPS4/HRH1/IGSF11/INF2/INPP5A/INPP5D/IQSEC1/IRS2/KCNIP1/KCNK10/KDM2B/LAMA4/LDB3/LIMD1/LRP1/LRP5/LRRC47/LSP1/MAD1L1/MAG/MAN1C1/MAP3K11/MAP4K4/MBP/MEGF6/MIDN/MOBP/MOG/MTSS1/MX1/MYO1F/MYO7A/MYT1L/NAV1/NCOR2/NDRG2/NDUFS2/NEU4/NFIX/NHSL1/NODAL/NOTCH4/NRXN3/NTM/NUAK1/OBSCN/ODF3L2/OLFML1/OPALIN/OPCML/P2RX7/PACRG/PCDH9/PDLIM2/PDLIM4/PEMT/PFKP/PHACTR1/PIK3R1/PRDM16/PRDM2/PRKCZ/PRR5L/PRRT1/PSD3/PTPRCAP/RAP1GAP2/RAPGEF5/RASA3/RASAL1/RBM47/RELL1/RFFL/RNF112/RNF208/RNF220/RPTOR/RTKN/RUNDC3A/RUNX1/RUNX3/SCARB1/SCG5/SCLY/SDCCAG8/SDK1/SEC31B/SGK1/SHROOM3/SKI/SLC38A10/SMURF1/SORBS3/SORCS2/SORT1/SOX10/SOX2OT/SPRED2/STK32C/STOX2/SYMPK/SYNJ2/SYNPO/THBS1/TJAP1/TMCC1/TMCC3/TMEM132B/TMEM88B/TOX2/TPPP3/TRAPPC9/TRIM2/TRIO/TSPAN32/TTBK1/TTC39C/TXNRD2/UNC13D/UNKL/WDFY4/WIPI2/ZAP70/ZBTB16/ZBTB38/ZC3H12D/ZDHHC14/ZFPM1/ZMIZ1/ |
| Hypermethylated in OPCs to OL transition | 161 | 1045 | 4.54E-37 | SEPT8/SEPT9/ABCG1/ABI3/ACSF3/ADAMTS2/ADARB2/ADORA2A/AFAP1/AGAP1/AGAP3/AJAP1/ANK3/ANKS1B/ARHGEF17/ASAP2/ATG16L2/ATP2A3/BANP/BCAT1/BCL11B/BTBD11/CACNA1C/CACNA2D4/CALD1/CAMK1D/CAMTA1/CAPN2/CASZ1/CCDC33/CCDC88C/CDH22/CDH4/CERK/CHN2/CHRM1/CLDN14/CMTM7/CNTFR/COL4A2/CPNE5/CRIP2/CTBP2/CUX1/CUX2/DNMT3A/DSCAM/EBF1/EBF3/EHD1/EMX2OS/ESRRG/FAM129A/FAM129C/FBLN2/FLI1/FOXP4/FRMD4A/GAD1/GALNT2/GPT2/GRAMD4/GRAP2/GRID1/GRIK4/HDAC4/HEPACAM/HIVEP3/HLCS/HOXB3/HPCAL1/HS3ST3B1/INPP5A/INPP5D/IQCE/JAK3/KALRN/KCNH2/KDM2B/KIRREL3/KREMEN2/LAG3/LASP1/LHX2/LHX6/LRBA/LRP1/LRP5/LSP1/LXN/MAB21L2/MAGI1/MAN1C1/MCF2L/MEGF6/MEIS1/MGAT5B/MPPED2/MTSS1/NCOR2/NDRG4/NFAM1/NFIX/NINJ2/NKX6-3/NOTCH4/NR2E1/OBSCN/OPCML/OTX1/P2RX7/PACRG/PAX6/PBX1/PCCA/PDLIM2/PDLIM4/PHACTR1/PIK3R1/PITPNM2/PLEKHO1/PRDM16/PRKAG2/RADIL/RFX4/RGMA/RGS10/RHPN1/RPTOR/RUNX1/RUNX3/SBNO2/SCUBE1/SDCCAG8/SDK1/SEMA5B/SFRP1/SH2D3C/SH3BP4/SHROOM3/SIX3/SLC1A5/SNED1/SORCS2/SPRED2/STK11/SYNJ2/TBC1D16/TMEM132B/TMEM132C/TNFRSF19/TOX2/TRIO/TRPM4/TTBK1/TTC22/VGLL4/WNT7B/ZBTB16/ZIC5/ZMIZ1/ |
| With H3K27me3 mark in OPCs | 53 | 551 | 7.14E-06 | ADAMTS2/ADORA2A/ATP2A3/ATXN1/B4GALNT1/BCL11B/BST2/CHST8/DHCR24/DHRS3/DNMT3L/ECEL1/ESRP2/FAM129C/FAM20C/FOXP4/FXYD1/GDF7/GIMAP1/GLP2R/GNAS/GPRC5C/GRB7/HSPA2/HTRA4/JAK3/KALRN/LOX/MAGI1/MID2/MYLK2/NODAL/PLCB2/PLCG2/PRDM1/PTK2B/RAB11FIP1/RASAL3/SCG5/SEC31B/SGK2/SLC22A18/SLC43A1/SORCS2/SPEG/SYMPK/SYTL1/TOX2/TP73/TRAPPC9/TRIM31/TTC22/WNT4/ |
| With H3K9me3 mark in iOLs | 58 | 647 | 2.41E-05 | ADARB2/ADD3/ARID5B/ATP6V0A4/ATXN1/BCL11B/CACNA2D4/CHST8/CMIP/CNTN1/DGKG/DHCR24/DMRTA2/EBF3/FAM20C/FGF2/GATA6/GPR81/GRID1/GRIK4/GRIN2B/HIVEP3/HOXA2/HOXA6/HSPA2/IGSF11/KIRREL3/LOX/LZTS1/MAP4K4/MTSS1/NINJ2/NKX6-1/NTM/OPCML/OTX1/PAX3/PBX1/PDE6A/POU2AF1/RAPGEF2/RGS14/RIN2/RUNX1/SCARB1/SH2D3C/SHANK2/SHROOM3/SIM2/SLC8A3/SMAD3/SPG20/SPRED2/TMCC3/TMEM132B/TNFRSF19/TRAPPC9/TRIM2/ |

**Additional file 5: Table S5**: Enrichment of the gene set: i) methylated genes in OPC to OL transition and ii) genes with H3K9me3 and H3K27me3 marks in OPCs and differentiating OLs; in XALD differentially methylated DMRs associated genes. Pathway, gene set definition; count, number of genes differentially methylated that are annotated at the gene set; size, number of genes from the 450 k Array that are annotated at the gene set, p-value, p values for each gene set tested by Fisher's exact test; genes associated, genes differentially methylated in XALD that overlap with the given gene set.
